# Spectral characterization of three wearable electronic ultraviolet radiation dosimeters

**DOI:** 10.1111/php.70085

**Published:** 2026-03-06

**Authors:** Leevi August, Gregor Hülsen, Juha Peltoniemi, Petri Kärhä, Erkki Ikonen

**Affiliations:** ^1^ Metrology Research Institute, Aalto University Espoo Finland; ^2^ Physikalisch‐Meteorologisches Observatorium Davos/World Radiation Center Davos Switzerland; ^3^ VTT Mikes Espoo Finland

**Keywords:** skin cancer, spectral mismatch, spectral responsivity, sunburn, UV detector, UV dosimeter, UV monitor

## Abstract

Excessive exposure to ultraviolet (UV) radiation poses significant public health risks, including DNA damage and skin‐related pathologies. This necessitates accurate studies and quantification of UV‐radiation exposure. Various wearable UV dosimeters have been developed to address these needs, particularly for outdoor workers. For measuring erythemal irradiance, recommendations from the World Meteorological Organization exist but standardized methods for characterization and calibration of wearable UV dosimeters are currently limited. At Aalto University and at Physikalisch‐Meteorologisches Observatorium Davos, three commercial electronic UV dosimeters were evaluated for their spectral responsivities using conventional measurement methods and traceability chains. The first device closely mimicked erythemal action in UV‐A and UV‐B regions. Two of the measured devices were found to have a higher relative responsivity of over 0.9 in the 300 nm to 310 nm range. Results were used to calculate correction factors for measuring erythemal radiance at varying solar zenith angles and ozone levels under cloud‐free conditions. Typical correction factors ranged from 0.98 to 1.2. The results of the two laboratories were in good agreement for the most accurate device but deviated due to the properties of the two other devices. The measurements revealed challenges posed by these devices in terms of data management, measurement times, and deployment, highlighting the need for standardized characterization methods.

## Graphical abstract

Excessive exposure to ultraviolet radiation poses significant public health risks, including DNA damage and skin‐related pathologies. Wearable electronic UV dosimeters enable accurate studies and quantification of solar UV‐radiation exposure on an individual level. Due to variations in the spectral distribution of solar radiation, the spectral mismatch between the dosimeters and erythemal radiation should be corrected to improve measurement accuracy. In this study, spectral responsivities of three commercial UV dosimeters were characterized for mismatch correction. Solutions for challenges posed by these devices, in terms of data management, deployment, and standardization of characterization, are considered and discussed.

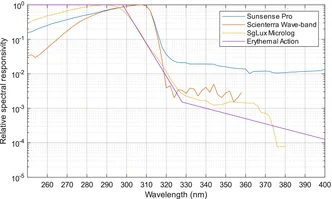

**Abbreviations**
CIE: International Commission on Illumination
DU: Dobson Unit
OMI: ozone monitoring instrument
SDM: standard deviation of the mean
SMCF: spectral mismatch correction factor
SRF: spectral responsivity function
SZA: solar zenith angle
TOC: total ozone column
UV: ultraviolet

## INTRODUCTION

Light significantly influences human health and well‐being through various mechanisms. Beyond its essential role in vision, light exposure has numerous non‐visual effects, such as circadian rhythm regulation and vitamin D synthesis.^1^, ^2^ Conversely, excessive exposure, particularly to ultraviolet (UV) radiation, carries various health risks, including DNA damage, eye damage, and skin‐related pathologies.^3^ Both beneficial and detrimental effects necessitate accurate studies and quantification of light and UV exposure.^4^

For non‐visual effects of visible light, the International Commission on Illumination (CIE) has introduced standard CIE S 026^5^ that defines spectral responsivity functions (SRFs), quantities, and metrics to describe various biological and physiological impacts of light. For UV exposure, standard ISO/CIE 17166^6^ defines erythemal dose and the erythemal action spectrum for weighting UV‐radiation. Erythema is also closely associated with other UV‐induced biological effects, such as DNA damage and skin aging.^7^, ^8^ For solar UV exposure, some safety recommendations exist. The most widely used guidelines are from the International Commission on Non‐Ionizing Radiation Protection^9^ and the American Conference of Governmental Industrial Hygienists.^10^ However, globally no standardized framework exists for quantifying exposure among outdoor workers, despite the significant occupational health risks associated with UV radiation.

To monitor and quantify UV exposure, various chemical,^11^ biological,^12^ and electronic^13^ UV dosimeters have been developed. Compared with model‐based estimates of solar radiation or UV radiation data from weather stations, dosimeters provide localized and temporally resolved measurements, enabling detailed analysis of either personal or environmental exposure.^14^ Electronic UV dosimeters enable real‐time monitoring and linking UV dosage to time and place by recording instantaneous UV intensities at predefined time intervals, e.g., every few seconds. This enables tying UV doses to time and location for health‐related research, as well as actively warning the wearer of high UV dosage and imminent sunburn. Such electronic UV dosimeters are small and lightweight, 5–50 g, and are attached to the body using a wrist or arm strap.

Most wearable UV dosimeters are designed to assess erythema risk, as sunburn represents the most immediate and quantifiable biological response to excessive UV‐radiation and correlates with other adverse effects. Therefore, the SRF of such devices would ideally match the CIE erythema action curve, which is also commonly used as a benchmark for harmful effects of UV radiation and is the basis for the UV index.^15^ A broadband response to UV radiation is necessary as the spectral distribution of sunlight varies significantly due to various factors.

The most influential factors defining the spectral distribution of solar radiation are the solar zenith angle (SZA) and the total ozone column (TOC). At larger SZA, sunlight travels a longer path through the atmosphere before reaching the ground than at small SZA. This substantially changes the ratio of scattered solar UV and direct sunlight, thus reducing spectral intensity of solar radiation in the UV region. TOC represents the amount of ozone in the atmosphere, and as ozone absorbs UV‐B radiation stronger than UV‐A, the spectrum changes as a function of TOC. SZA can be calculated from location and time, and TOC can be predicted with various models. TOC can also be acquired from ground or satellite measurements, such as the ozone monitoring instrument (OMI)^16^ or European Brewer Network (Eubrewnet).^17^ During field measurements, both SZA and TOC affect measurements with dosimeters at all times, making their contribution substantial but predictable.

The solar spectral distribution is also influenced by various localized or dynamic factors. Aerosols, such as cloud cover,^18^, ^19^ cause spectral scattering, and atmospheric pollution can absorb radiation in specific wavelength regions. For example, photons of UV‐B radiation are absorbed by ozone and sulfur dioxide, while photons of UV‐A radiation are absorbed most by nitrogen dioxide.^20^ Albedo of the environment, such as water, snow, sand, or buildings, can also have a different spectral profile from the incident sunlight. Likewise, the changes in the balance of scattered and direct sunlight in urban environments can change spectral distribution.^21^ These factors cause unpredictable but temporary fluctuations in the solar spectra.

For typical solar irradiance, the vast majority of the erythemal dose is contributed by wavelengths longer than 290 nm, as shorter wavelengths have very low spectral intensities due to atmospheric absorption.^22^ The largest relative contribution to erythemal dose is from 300 to 320 nm wavelengths, making this region of UV‐B of particular interest. This is also the region most affected by fluctuations in the spectral distribution of solar radiation.

Broadband solar UV dosimeters need calibration to measure UV doses accurately. Calibrations can be carried out under the Sun or using artificial light sources. Spectral responsivity characterizations of UV dosimeters are required to account for errors due to spectral mismatch of the light sources used in calibration and the solar spectra at the time of measurement. While laboratory‐based methods for spectral measurement and light‐source characterization are well‐established, wearable devices present unique challenges. Designed for field conditions, these devices must balance portability, affordability, and ease of use, often at the expense of precision and transparency of typical laboratory instruments. Although such devices have been characterized in the past^23^, ^24^ the absence of standardization complicates the assessment of device accuracy and the traceability of their measurements. However, general recommendations for measuring erythemally weighted broadband radiation have been given by the scientific advisory group on ozone and solar UV radiation of the World Meteorological Organization. This is best summarized in a practical guide by Webb et al.^25^

In this work, three readily available commercial wearable electronic UV dosimeters are characterized for spectral responsivities and a method for correcting spectral mismatch is presented. The spectral characterization of the UV dosimeters was carried out at the Metrology Research Institute of Aalto University (Aalto) and at the World Radiation Center of Physikalisch‐Meteorologisches Observatorium Davos (Davos). The work is in the context of a larger project of the European Union called Metrology for Wearable Light Loggers and Optical Radiation Dosimeters (MeLiDos). Devices were measured for spectral responsivity with uncertainty evaluation and further converted to correction factors for various scenarios of SZA and TOC.

## MATERIALS AND METHODS

### Characterized devices

**FIGURE 1:**
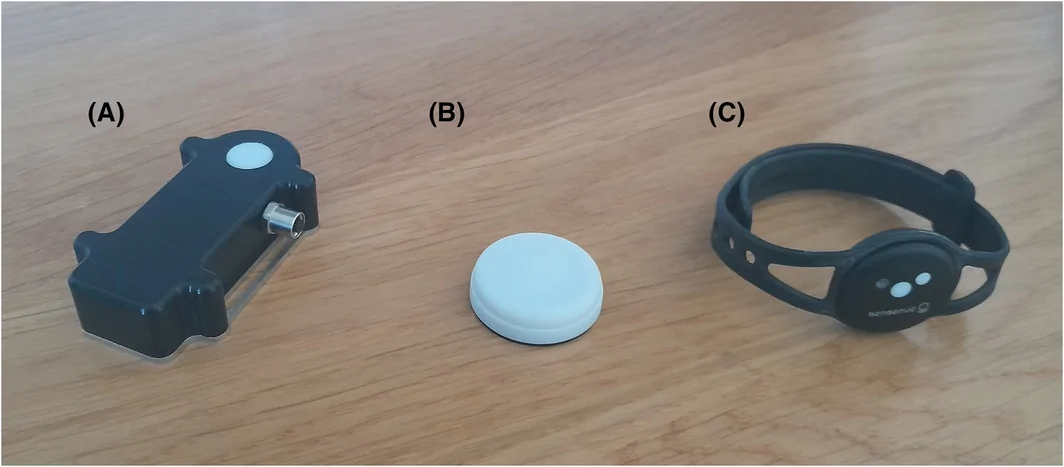
(A) SgLux UVMicrolog, (B) Scienterra Wave‐band, and (C) Sunsense‐pro dosimeters characterized in this study.

Three commercially available wearable UV dosimeters were selected for this study: UVMicrolog produced in Germany by SgLux,^26^ Wave‐band produced in New Zeeland by Scienterra,^27^ and Sunsense‐Pro produced in Norway by Sunsense AS.^28^ These devices, shown in Figure 1, represent the current readily available selection of electronic dosimeters for personal UV‐radiation exposure. Several comparable instruments have previously existed but are no longer in production, likely due to limited demand or availability of key components.

All three dosimeters are designed for the consumer market and aim to quantify the personal UV exposure of individual users. Each device employs photodiode‐based detectors, in which the incident UV radiation induces an electrical current proportional to the received irradiance. The final spectral responsivity of each dosimeter is determined by the intrinsic responsivity of the photodiode, the optical transmission of diffusers and filters, and potentially altered by undocumented internal procedures.

UVMicrolog is initialized using a control software while connected to a computer via a USB interface. The control software allows for calibrating the device and adjusting various settings such as sampling frequency as a part of initialization, after which the device operates independently. In addition to its primary photodiode readings of erythemally weighted UV irradiance, the device records temperature and broadband white light. Data are saved on the device as a log with time and date, and the full raw data are available to download and export using the control software. The device dimensions are 59 mm × 33 mm × 16 mm with a weight of 40 g.

Wave‐band device is still in development, but it has several preceding models which have been successfully deployed. It currently requires a constant serial connection to a computer from which it is operated. A firmware update is in development to allow for Bluetooth connection to the device, enabling measurements without a physical connection, but was not available during this study. Wave‐band features a primary UV‐B channel for measuring erythemal irradiance. The device also includes optical channels for UV‐A, near infrared radiation, red, green, and blue light as well as sensors for temperature and acceleration. The device streams raw data from all sensors including UV‐B data with various applied corrections which can be recorded using supporting software. The device has a radius of 14 mm and a thickness of 6 mm, with a weight of 10 g.

Sunsense‐pro connects to a mobile device through Bluetooth connection and can be activated by manually tapping the device. The settings and calibration of the device are inaccessible to a regular user, and the sampling frequency changes dynamically based on various factors. The device has a primary UV‐B radiation channel and includes secondary channels for red, green, and blue light, as well as a temperature sensor. No data are saved on the device but are instead streamed to the hosting mobile device, where an operating app saves them as a .csv file. The device has a radius of 13 mm and a thickness of 7 mm with a weight of 8 g.

During characterization measurements, several challenging features of the dosimeters were identified. At Davos, the angular responsivities of the devices were measured and found to deviate from cosine response. This does not change the result for spectral responsivity characterization at perpendicular incidence but must be accounted for when calculating spectral mismatch correction as the angular responsivity affects the ratio of direct and diffuse sunlight. Temperature dependence is significant in the designed application of the devices, as they are intended to be worn by users in outdoor conditions, where the temperature can range from −20 to +50°C. However, in laboratory measurements, temperature can be controlled within 1–2°C.

During testing, each device was noted to exhibit hysteresis, which was particularly influential for the measurements of Sunsense‐pro and Wave‐Band dosimeters. For the intended use of the device, measuring integrated doses over a day, this is typically not a meaningful source of uncertainty. However, for spectral measurements in laboratory conditions, this requires allocated rise and fall times for signals and dedicated periods for dark measurements. To test hysteresis in the dosimeters, the devices were exposed to radiation from a monochromator output set at the wavelength of 300 nm, known to induce a high reading in the dosimeters. After the counts for the tested device had settled, the exposure was changed to 270 nm interspersed with dark signal.

For Wave‐band, the initial signal at 300 nm took 1.5 min to settle and resulted in 3100 counts/s. After changing the wavelength to 270 nm, an initial reading of 1060 counts/s slowly began to decline. During the decline, the radiation was cut for 3 s, and typical dark readings of the dosimeter were noted. After the dark period the signal remained elevated and continued to decline to a level of 750 counts/s over 2 min. After the signal stabilized, the radiation was cut again and allowed to remain dark for 45 s. The low signal was restored and the readings from the dosimeter started at 520 counts/s and began to recover over 40 s toward the 750 counts/s, where the signal previously stabilized. The test showed that the dosimeter increased or decreased its responsivity based on prior exposure, and the effect was particularly notable when moving from long to short wavelengths. A 30‐s radiation time was found to be sufficient to clear the hysteresis when moving in 1 nm steps. Similar behavior was noted in Sunsense‐pro, but longer internal integration times seem to limit the effect. Some hysteresis also was noted in UVMicrolog, but this typically cleared within 5 s.

The dosimeters log data in various ways, independently of the operator. For Sunsense‐pro, logging takes place with varying frequencies. This makes pairing data from the UV dosimeters and the reference device challenging. Additionally, specific designs of the enclosures and sensor location in some devices introduced mechanical challenges during alignment. Several methods were independently developed by members of the project to resolve these issues. At Aalto, data were paired with reference values by adding timestamps for each measured wavelength, and timestamps were synchronized with short periods of continuous bright exposure. Additionally, devices that store data externally can easily lose measurement data if the connection to the external device is disrupted or not correctly initialized. Lack of continuous monitoring of the connection state and data storage further complicates data management.

### Measurement methods and setups

The selected UV dosimeters were measured for spectral responsivities at two laboratories, Aalto and Davos. Measurements were made at two different laboratories to compare methodologies and to identify possible systematic sources of deviations caused by nonobvious factors like hysteresis. Other parameters such as temperature dependence and cosine response were measured and will be reported separately.

**FIGURE 2:**
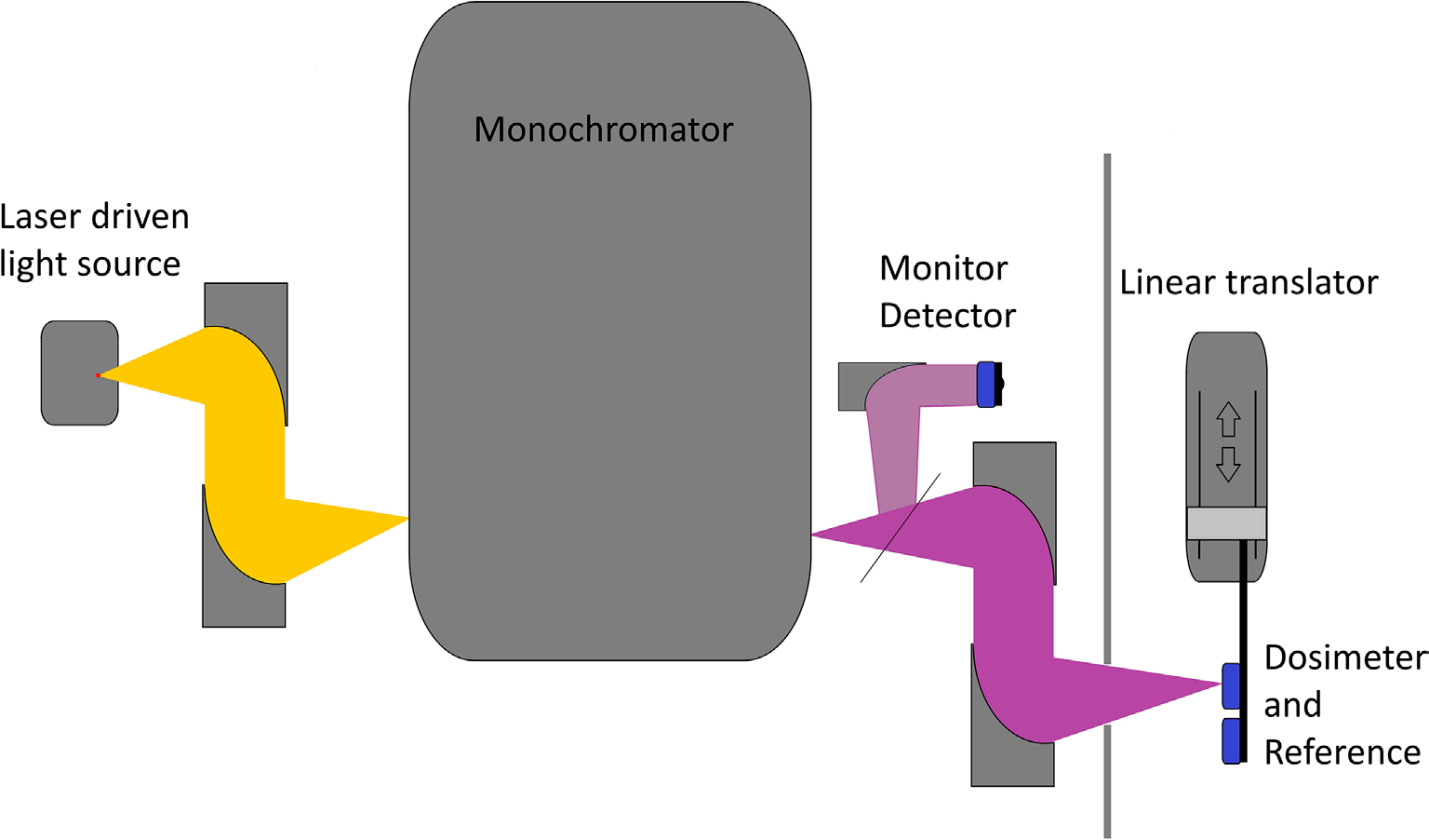
Measurement setup at Aalto University for spectral responsivity characterization.

At Aalto University, the measurements of relative spectral power responsivities were made with a reference spectrometer setup using a substitution comparison with a reference detector. The setup, presented in Figure 2, has been developed and characterized at Aalto University.^29^ A diffraction grating double‐monochromator and order sorting filters are used to select a wavelength of the primary radiation source, a laser driven xenon plasma light source, EQ‐99X‐QZ‐S. The Princeton SP‐2760 monochromator incorporating three diffraction gratings and diffraction order filters provides scanning for wavelengths from 220 to 2300 nm. Output from the monochromator is focused into a 2 mm wide square beam and directed onto a measurement stage, which allows for switching between illuminating a measured detector and a reference detector by moving the detectors in or out of the beam using linear translators. The reference used was an S‐2281 Hamamatsu silicon detector amplified by a current‐to‐voltage converter and read with a digital multimeter. A triangular bandwidth, with full width at half maximum of 2 nm, was used in the measurements. The room temperature for the measurements was (21.7 ± 0.7)°C.

The setup has a monitor detector to which a fraction of the light is directed from the monochromator output using a beam splitter. The monitor detector is typically used to correct for potential power fluctuations between measuring the target device and measuring the signal with the reference. However, the dosimeters cannot be integrated into the measurement program of the setup; thus, there is no way of synchronizing the trigger for the monitor with the measurement points of the dosimeters. This prevents the monitor from being used for correcting minor fluctuations which will instead present as uncertainty in the standard deviation of measurement points.

The dosimeters were measured from 250 to 400 nm with a step of 1 nm, starting from short wavelengths. The reference detector and the diffusers of the dosimeters were underfilled. However, as the diodes within the dosimeters are smaller than the diffusers, the results are not representative for the whole diffuser area in absolute values. This is particularly relevant for Wave‐band, which has the entire front plane of the device covered by a diffuser, but was also noticed in UVMicrolog.

For each wavelength step, the dosimeters were radiated for a 30‐s period to clear hysteresis followed by a 20‐s period for taking measurement readings. For Wave‐Band, the measurement period was 10 s. The final value for each wavelength was integrated from these readings. Additionally, a 10‐s dark period was used to acquire dark values for the dosimeters and reference. During analysis, it was determined that the dark values for Sunsense‐pro did not recover in the allotted time, and the device was remeasured using dark values from a 5‐min dark period before and after the measurement.

At Davos, the spectral characterization was made using a calibrated quasi‐monochromatic light source with a known relative spectral distribution. The light source is based on a Princeton Instruments SP255 double monochromator and a 450 W xenon lamp as the primary radiation source. The radiation has a full width at half‐maximum bandwidth of 2 nm and an approximately triangular slit function. Behind the exit slit, a quartz plate mounted at 45° vertically transmits about 92% of the radiation toward the test detector, while about 8% of the radiation is deflected toward a photodiode, which is used to monitor the stability of the monochromator output signal. The spectral responsivity of the system is realized by the TRAP diode PMOD/WRC #1, traceable to the Eidgenössisches Institut für Metrologie METAS, Switzerland. A more complete description of the measurement setup can be found in previous publications.^30^

The beam profile of the monochromator light source is square flat‐top with a diagonal of 17 mm, which results in an underfilling of the detector diffuser of Wave‐Band and overfilling the diffusers for Sunsense‐pro and UVMicrolog. SRFs of the characterized dosimeters were obtained by illuminating them with the monochromatic light source and recording the measured signal. The SRFs were measured from 270 to 420 nm, descending from 400 nm with a step of 2 nm and normalized to the value obtained at maximum responsivity. Measurement times per wavelength were 20 s for Wave‐band, 10 s for UVMicrolog, and 40 s for Sunsense‐pro. The room temperature for the measurement was (22.5 ± 0.3)°C.

### Uncertainty budgets

For the uncertainty analysis, we consider the uncertainties of the reference detector, the spectrometer setups and the measured devices. The expanded uncertainties for the measurements at Aalto University are presented in Table 1. The calibration of the reference detector Hamamatsu S‐2281 is traceable to national standards via a pyroelectric radiometer. Expanded uncertainty (*k* = 2) for the reference detector S‐2281 is 2% at 380 nm, increasing nearly linearly to 5% at 250 nm.

**TABLE 1:** Typical relative expanded uncertainties (*k* = 2) of the spectral responsivity characterization at Aalto for UVMicrolog, Wave‐band, and Sunsense‐pro UV dosimeters in each UV region.

Device	UVMicrolog (%)	Wave‐Band (%)	Sunsense‐pro (%)
UV‐C < 280 nm	5	5	5
UV‐B, 280–315 nm	4	4	4
UV‐A, 315–400 nm	13	90	8

According to the prior characterization of the setup, drift of the monochromator output is smaller than 0.1% between measuring dosimeter signal and switching to reference. Stray light within the measurement setup is estimated to cause a relative uncertainty of 0.1%. Wavelength uncertainty of the monochromator setup is 0.05 nm corresponding to a maximum relative uncertainty of 3.7% in relative responsivity. The uncertainties due to electrical amplification of the reference detector signal and the digital voltmeter calibration are negligible as compared to the other sources.

Relative standard deviation of the mean (SDM) for repeated measurements varies per detector and wavelength. For all devices, SDM increases in UV‐A as most of the responsivity of the devices is in UV‐B. At very low counts, less than 10, SDM does not properly represent uncertainty. At these counts, the signal could change several percents without being sufficient to change the number of counts. In such regions, a minimum of 5% uncertainty from SDM was allocated and a minimum of 10% for readings below 5 counts.

SDM for UVMicrolog is smaller than 1% in UV‐C and UV‐B regions. In UV‐A, the low signal levels cause noise to become significant, increasing SDM from 1.5% at 315 nm to 24% at 360 nm, but typically remaining smaller than 12%. For the final wavelengths with signal, 373–380 nm, the relative uncertainty rapidly increases from 10% to 134% as the signal is lost.

SDM for Wave‐Band is up to 17% for wavelengths shorter than 260 nm. For the rest of UV‐C it is smaller than 4% and in UV‐B smaller than 1%. Due to low responsivity in UV‐A and a noisy signal from the device, the signal‐to‐noise ratio for Wave‐Band in UV‐A is smaller than 1 and cannot be determined without the integrated values. The high noise causes SDM to quickly rise from 1% to a range typically between 50% and 120% and 4 values reaching around 200%.

SDM for Sunsense‐pro is smaller than 2% in UV‐C, and smaller than 1% in UV‐B. In UV‐A, SDM is smaller than 3%, but at most wavelengths longer than 324 nm, the 5% and 10% limits were used due to the low signal.

For the measurements at Davos, the expanded uncertainties (*k* = 2) for the spectral responsivities of the dosimeters are 5%–6% for UVMicrolog, 5%–30% for Wave‐Band, and 5%–9% for Sunsense‐pro, depending on wavelength as presented in Table 2.

**TABLE 2:** Typical relative expanded uncertainties (*k* = 2) of the spectral responsivity characterization at Davos for UVMicrolog, Wave‐band, and Sunsense‐pro UV dosimeters in each UV region.

Device	UVMicrolog (%)	Wave‐Band (%)	Sunsense‐pro (%)
UV‐C < 280 nm	5	5	5
UV‐B, 280–315 nm	6	6	7
UV‐A, 315–400 nm	6	30	9

## RESULTS

### Spectral responsivity

**FIGURE 3:**
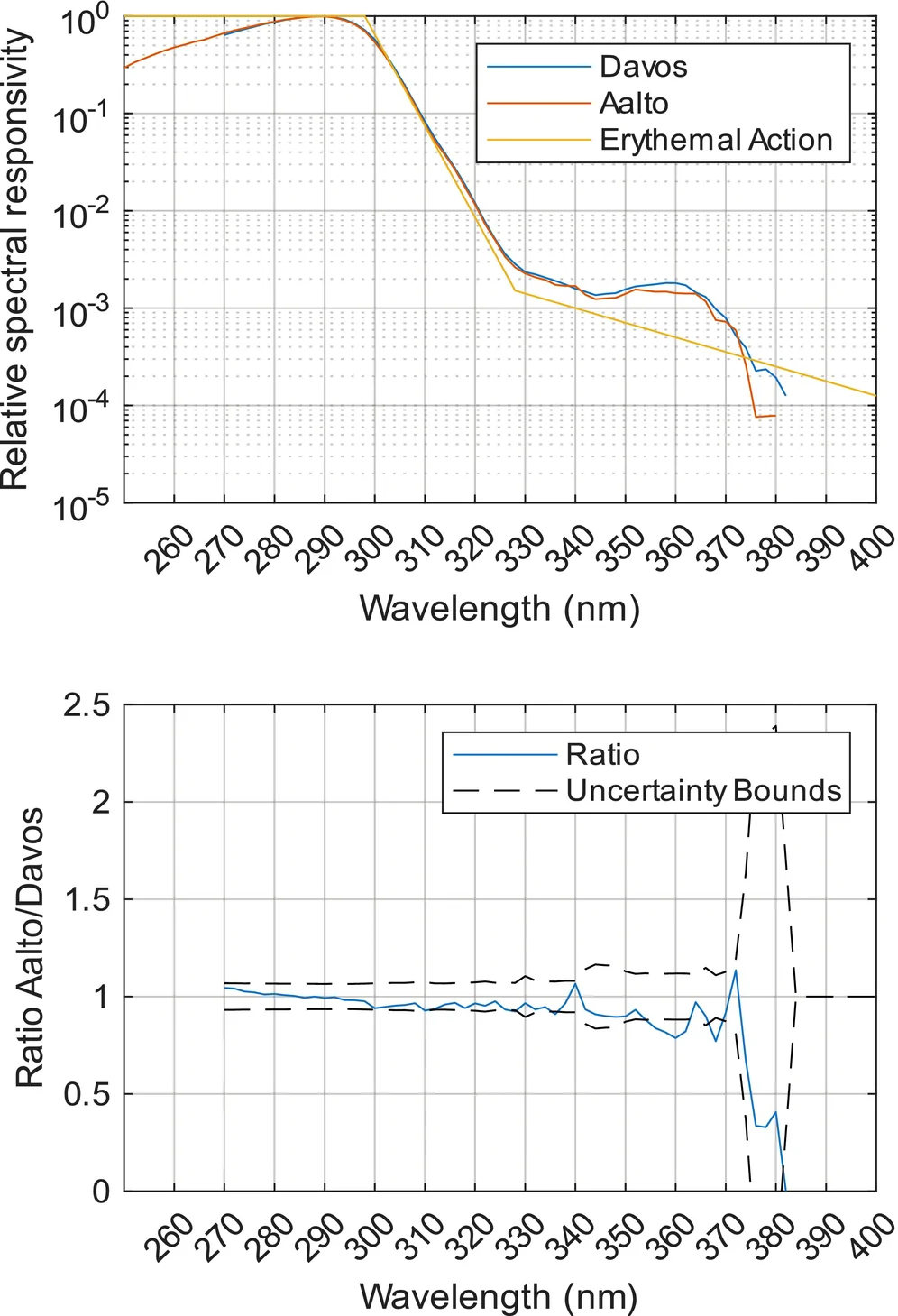
Spectral responsivity of UVMicrolog as measured at Aalto and Davos. The ratio of the two measurements is shown in the bottom half of the figure, together with upper and lower bounds from the combined expanded uncertainty (*k* = 2) of the participants.

**FIGURE 4:**
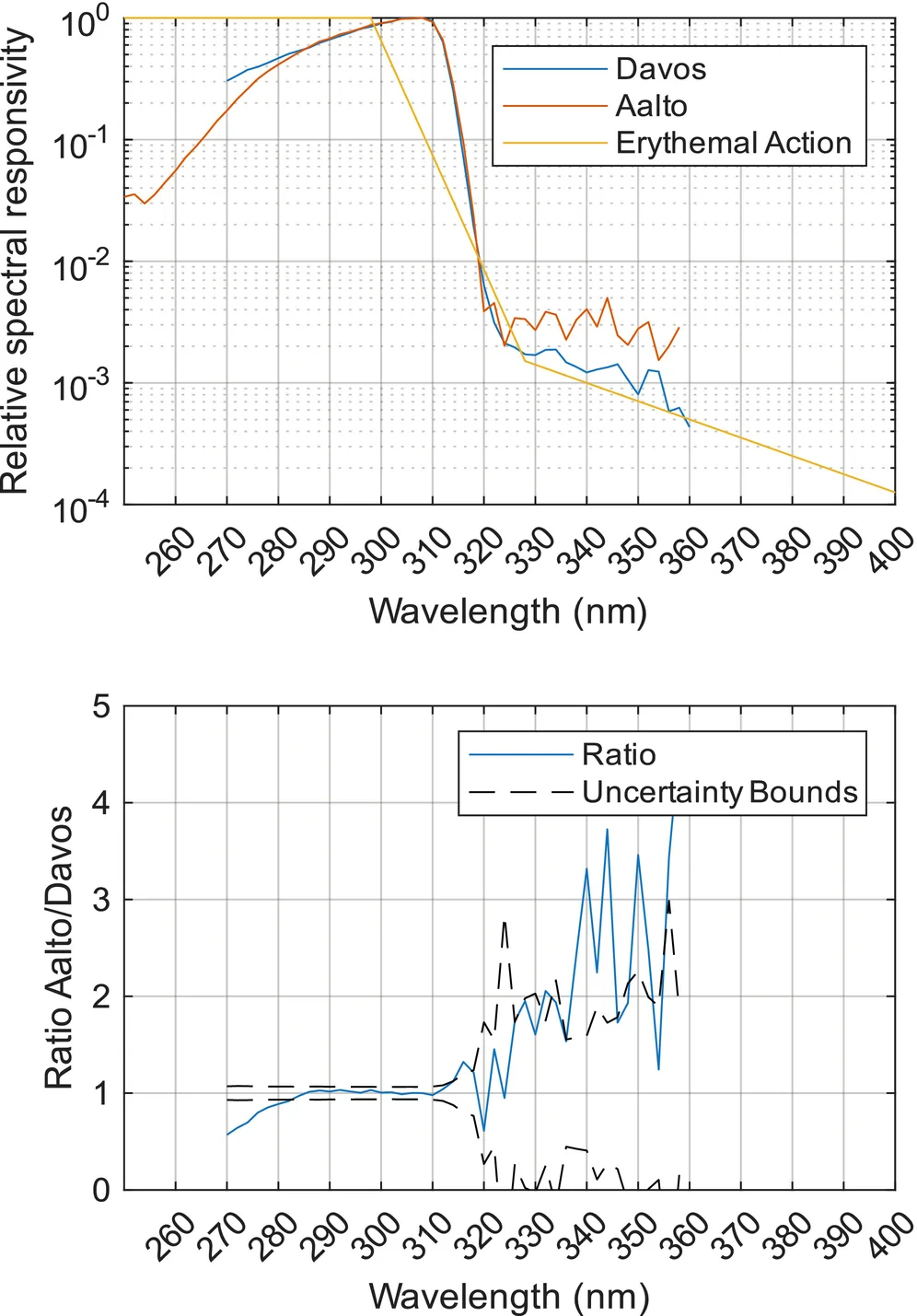
Spectral responsivity of Wave‐band as measured at Aalto and Davos. The ratio of the two measurements is shown in the bottom half of the figure, together with upper and lower bounds from the combined expanded uncertainty (*k* = 2) of the participants.

**FIGURE 5:**
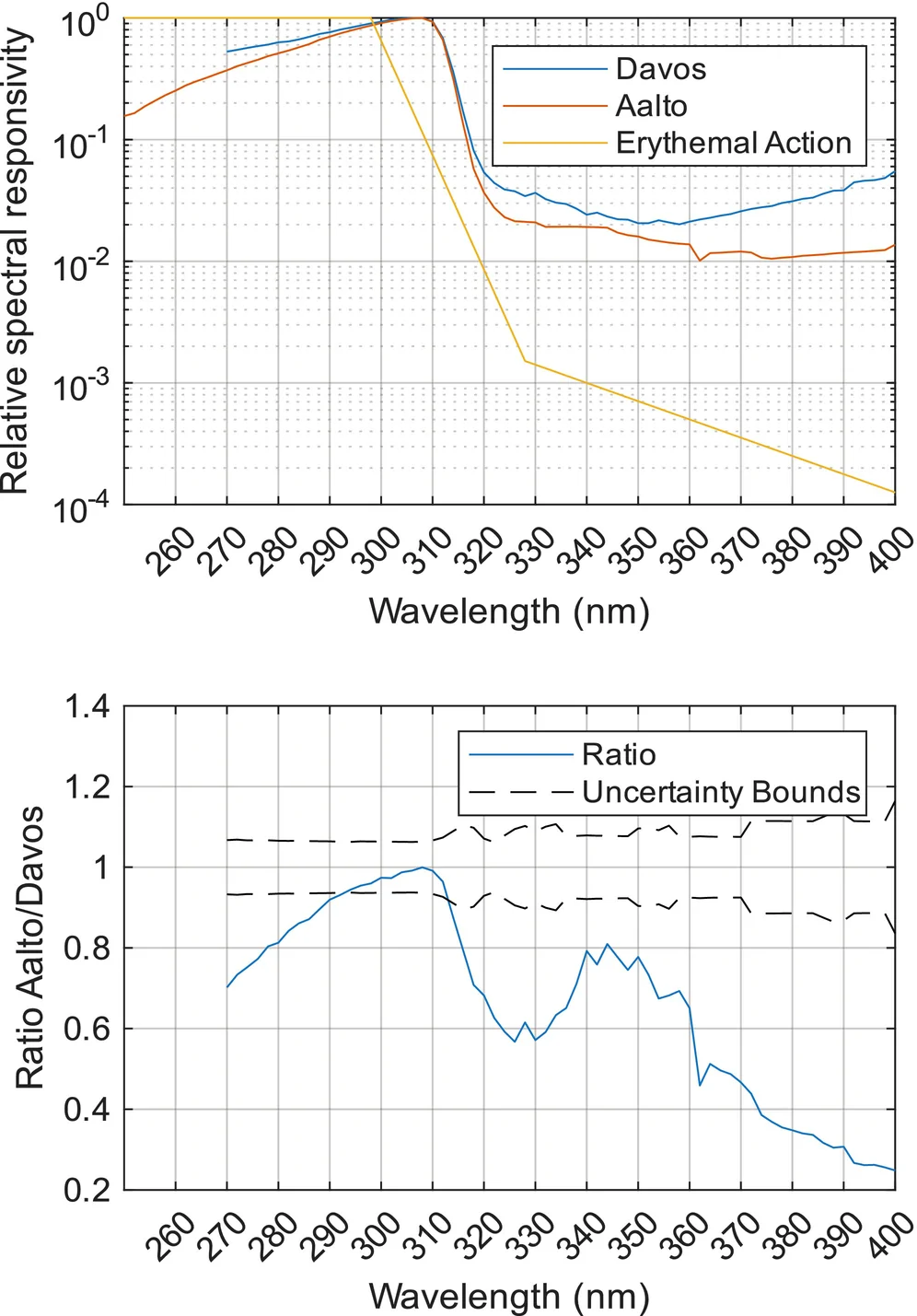
Spectral responsivity of Sunsense‐Pro as measured at Aalto and Davos. The ratio of the two measurements is shown in the bottom half of the figure, together with upper and lower bounds from the combined expanded uncertainty (*k* = 2) of the participants.

The normalized spectral responsivities of the UV dosimeters were compared with the CIE erythemal action spectrum,^6^ which is considered the ideal spectral weight for the purposes of this characterization. The results measured at Aalto and Davos are compared with each other to assess the reproducibility of the spectral characterization results and possible differences in measurement setups. The results are presented in Figures 3, 4, 5, where the uncertainty bounds are Root‐Sum‐Squares of the Aalto and Davos measurement uncertainties.

All three dosimeters exhibit between 5% and 50% relative responsivity in the UV‐C region. While these wavelengths are not present in natural sunlight due to complete atmospheric absorption, their presence must be considered when artificial UV sources are used for calibration or characterization.

As seen in Figure 3, the measurements of Aalto and Davos for UVMicrolog were generally in good agreement and within uncertainties. According to the measurements, UVMicrolog exhibits a spectral responsivity that closely follows the erythema action spectrum across the UV‐B region. Deviations of 5%–30% from the erythemal action spectrum were observed from 294 to 300 nm. In UV‐A below 375 nm wavelengths, UVMicrolog has a two to four times higher relative responsivity as compared to the erythemal action spectrum. For wavelengths longer than 380 nm, the device gives little to no signal. Overall, its close alignment indicates good spectral weighting for erythema‐related UV monitoring.

Measurements of Aalto and Davos for Wave‐Band, as seen in Figure 4, are in agreement with each other in UV‐B. In UV‐C, Aalto measured 13%–40% lower responsivities, with the deviation increasing toward shorter wavelengths. This is likely due to the hysteresis discussed in characterized devices. Davos measured from long wavelengths to short ones at 10 s per wavelength, whereas Aalto measured from short wavelengths to long. An attempt to replicate the effect at Aalto, by measuring UV‐B and UV‐C in a similar manner to Davos, resulted in a 30% higher responsivity at the wavelength of 270 nm. This explains the difference between Aalto and Davos in Figure 4 at around 270 nm. In UV‐A, the responsivities measured by Aalto are two to three times higher than those measured at Davos. However, measurement results for Aalto become unreliable in UV‐A, as low signal to noise ratio causes uncertainty to grow over 100%. The measurement results indicate that the Wave‐Band has responsivities up to 11 times higher than the erythemal action spectrum in the wavelength region from 300 to 318 nm, and little to no response for wavelengths longer than 350 nm.

For Sunsense‐pro, Aalto and Davos acquired the same general shape of SRF, but Davos consistently measured higher responsivities throughout the wavelength ranges. The results are within their combined uncertainties from 290 to 315 nm. A difference from 10% to 35% can be seen at wavelengths shorter than 290 nm and 10% to 75% in UV‐A. The exact reason for the difference is unknown, but a possible explanation could be device nonlinearity. The intensity level used at Davos was higher than the intensity available at Aalto. The difference in relative signal between UV‐A and UV‐B is reduced if the dosimeter saturates. The responsivity in the wavelength region 300–320 nm is up to 11 times higher than the erythemal action spectrum, as well as 10 to 20 times higher in the UV‐A region.

### Spectral mismatch correction

Based on the measurement results for the SRF of the dosimeters, a correction factor is required to correct for the mismatch between erythemal action and the spectral responsivities of the characterized UV dosimeters. As the spectral distribution of solar radiation varies due to several factors, a single correction factor is insufficient. Likewise, correcting for all fluctuations is unfeasible in practice as it would require complete knowledge of all environmental factors. However, the most influential factors of SZA and TOC can be evaluated. Zenith angle can directly be calculated from location and time, and model predictions and weather data for ozone columns are widely available. Using modeled SZA and TOC, the spectral distribution of solar radiation can be modeled and then used for calculating spectral mismatch under clear sky conditions.

The spectral mismatch correction factors (SMCF) for each dosimeter can be calculated from the spectral responsivity of the dosimeter *S*_rel_ $\left(\lambda \right)$, ideal erythemal action spectrum *A*_act_ $\left(\lambda \right)$, relative spectral distribution of light used for calibration $X_{\lambda ,R,\mathrm{rel}}\left(\lambda \right)$, and the relative spectral distribution of measured light $X_{\lambda ,Z,\mathrm{rel}}\left(\lambda \right)$ using equation

(1) $$\begin{array}{c} \text{SMCF}=\frac{\int X_{\lambda ,R,\mathrm{rel}}\left(\lambda \right)A_{\mathrm{act}}\left(\lambda \right)d\lambda }{\int X_{\lambda ,R,\mathrm{rel}}\left(\lambda \right)S_{\mathrm{rel}}\left(\lambda \right)d\lambda }\times \frac{\int X_{\lambda ,Z,\mathrm{rel}}\left(\lambda \right)S_{\mathrm{rel}}\left(\lambda \right)d\lambda }{\int X_{\lambda ,Z,\mathrm{rel}}\left(\lambda \right)A_{\mathrm{act}}\left(\lambda \right)d\lambda } \end{array}$$

Integration limits correspond to the erythemal action wavelength range of 250 nm to 400 nm. The resulting correction factors can be collected into a correction matrix as presented for the Wave‐Band dosimeter in Table 3, using the SRF measured at Davos and simulated solar spectra for various SZA and TOC values, normalized to TOC 300 DU (Dobson units), and SZA 40°.

**TABLE 3:** Correction table of Wave‐Band dosimeter for solar zenith angle (SZA) and total ozone column (TOC) based on the spectral responsivity measurements at Davos. The table is normalized to the relative spectral distribution at TOC 300 DU, SZA 40**°**.

SZA\TOC	220 DU	260 DU	300 DU	340 DU	380 DU	420 DU	460 DU	500 DU
0°	1.141	1.078	1.037	1.011	0.998	0.993	0.996	1.006
5°	1.139	1.077	1.036	1.011	0.998	0.994	0.997	1.006
10°	1.135	1.073	1.033	1.009	0.997	0.994	0.998	1.008
15°	1.127	1.067	1.029	1.007	0.996	0.994	1.000	1.012
20°	1.116	1.059	1.023	1.004	0.995	0.996	1.004	1.018
25°	1.102	1.049	1.017	1.000	0.995	0.999	1.010	1.028
30°	1.086	1.038	1.010	0.998	0.997	1.005	1.020	1.042
35°	1.069	1.026	1.004	0.997	1.001	1.014	1.035	1.062
40°	1.050	1.015	1.000	1.000	1.010	1.030	1.058	1.093
45°	1.033	1.006	1.000	1.008	1.027	1.056	1.093	1.137
50°	1.017	1.002	1.006	1.025	1.056	1.096	1.145	1.203
55°	1.007	1.006	1.024	1.057	1.103	1.160	1.226	1.303
60°	1.007	1.024	1.061	1.114	1.181	1.261	1.353	1.458
65°	1.024	1.067	1.131	1.213	1.312	1.428	1.559	1.707
70°	1.074	1.154	1.258	1.386	1.537	1.710	1.906	2.125
75°	1.186	1.324	1.496	1.701	1.939	2.209	2.513	2.851
80°	1.424	1.661	1.946	2.278	2.657	3.081	3.548	4.057
85°	1.865	2.224	2.634	3.093	3.597	4.145	4.734	5.362
90°	1.948	2.224	2.538	2.890	3.281	3.712	4.183	4.695

## DISCUSSION

The spectral responsivity characterization results indicate the need for spectral mismatch corrections for the measured devices and highlight the need for UV dosimeters to be thoroughly characterized. Corrections for spectral mismatch can be implemented within the software of a device if it has access to the required data. A simplified correction can be made from location and time by assuming a TOC value for the region and time of year. If real‐time feedback is not required, mismatch corrections can also be applied in postprocessing using a known SRF or a SZA/TOC correction table provided by the manufacturer or supplier.

Spectral responsivity characterization is highly time‐consuming and requires specialized equipment. For devices with dynamic sampling frequency or high hysteresis, measurement at a single wavelength can take several minutes, so obtaining data for the full UV region takes several hours. Additionally, devices can have multiple channels for different wavelength regions, complicating characterization further. Therefore, such characterization is not appropriate for typical end users to perform. It is also not practical to measure it for each individual device, but rather once for a device type or production batch.

Once corrected for spectral mismatch, each device in this study can be expected to perform erythemally weighted measurements in most conditions. However, in conditions where the device and its wearer are exposed to radiation that does not follow solar distribution, spectral mismatch can still have substantial effects. Aerosols, most notably clouds, can change the spectral distribution through absorption or reflection. Albedo can also have a significant effect in aquatic, snowy or sandy environments. These can be accounted for as additional measurement uncertainty but could be modeled and corrected for, similar to SZA/TOC, if sufficient data are available. For certain professions, such as truck or bus drivers, practitioners are likely to be exposed to several hours of sunlight through glass windows which typically exhibit low UV‐B transmittance.^31^ People exposed to artificial light sources may also experience significant doses of artificial UV radiation which differs from solar spectrum. Such sources include plant lights, UV‐curing stations for various UV‐activated materials and some disco lights. If one is investigating the occupational risk posed to the health of such individuals, effects of spectral mismatch should be accounted for.

Using an auxiliary UV‐A detector allows devices to present readings in atypical measurement conditions. As it has been shown that the erythemal action curve does not fully represent the extent of cutaneous damage or environmental effects, dosimeters used for medical research or environmental monitoring may benefit from independent channels in UV‐A or visible wavelengths.

Based on the measurements, several recommendations can be made for the spectral characterization of UV dosimeters. When using artificial sources, such as xenon lamps, it is important to consider the spectral distribution of these sources. The high intensity radiation of these sources in the UV‐B region can oversaturate detectors designed for solar radiation, which was perceived during some of the early testing and likely in the results presented in Figure 5. Comparison measurement results could be improved by ensuring that measurements are made using the same power level. Furthermore, the sequence of measurements could be unified with either ascending or descending wavelength steps.

In a typical spectral laboratory comparison between detectors, results are read out in real time via voltage or current terminals. This cannot be done with dosimeters as the devices measure on either set time intervals or use dynamic measurement rates determined by the device. Data are also not readily available but must be recovered from a storage file separately. This causes synchronization issues with matching measured data from the dosimeters to the data from the reference detector. One should make sure that both datasets have adequate time stamps to combine the data. A recorded signal for synchronizing the time stamps may also be necessary as the internal clocks of the devices may not be up to date. Test runs with step changes in power levels should be made to determine the devices for complicating features such as hysteresis and behavior of dark values.

To reduce the time required for spectral characterization, a simplified method for spectral characterization could include measuring dosimeters at a selection of 4–6 specific wavelengths to acquire a general shape for the SRF. Wavelengths of 290, 300, and 330 nm are good candidates for casting the slope of erythemal action. Wavelengths 313 and 365 nm are commonly used in other applications; therefore, they are more readily available and complement the other wavelengths well.

It is worth noting that in practical use, a significant source of uncertainty for wearable UV dosimeters comes from the wearing habits and behavior of the subject wearing such a device. As a result, the devices must compromise measurement accuracy, size and wearability, as well as cost in their design. Practical applications may also be limited by other factors such as data protection issues regarding location, thus limiting the use of data processing based on locational data such as ozone levels or SZA.

## CONCLUSIONS

Three commercially available wearable electronic UV dosimeters were measured at Aalto University and PMOD/WRC Davos for spectral responsivity. The variation of spectral distribution of solar radiation necessitates correcting for spectral mismatch between the ideal erythemal action spectrum and the SRF of UV dosimeters used to measure solar radiation. Correction factors for spectral mismatch were calculated using simulated solar radiation at various zenith angles and ozone conditions. Typical correction factors ranged from 0.98 to 1.2. However, at SZAs greater than 70 degrees, for example the first 2 h after sunrise or before sunset, the correction factor could reach as high as 5.3 in high atmospheric ozone conditions. Although UV irradiance is low for SZA greater than 70°, its effect can be significant, particularly in northern regions during winter.

Varied non‐standardized calibration methods result in potential inaccuracies in measuring UV. For example, the UV‐C sensitivity of the dosimeters must be rigorously considered when using artificial light sources, such as xenon arc lamps, for calibration. Failure to account for spectral sensitivity could lead to erroneous readings and misguided safety measures. Moreover, the effects of temperature dependence and angular responsivity when used in diverse outdoor conditions necessitate development of robust performance indicators. The extensive duration and complexity involved in characterizing UV dosimeters underscore the need for a simplified and standardized characterization method, particularly for spectral responsivity, when calibrating large quantities of these devices. Finally, special attention should be given to properly account for the effects of human behavior and equipment use when estimating the end‐user UV‐dose.

## Data Availability

The data that support the findings of this study are available from the corresponding author upon reasonable request.
